# A deep-sea hydrothermal vent worm detoxifies arsenic and sulfur by intracellular biomineralization of orpiment (As_2_S_3_)

**DOI:** 10.1371/journal.pbio.3003291

**Published:** 2025-08-26

**Authors:** Hao Wang, Lei Cao, Huan Zhang, Zhaoshan Zhong, Li Zhou, Chao Lian, Xiaocheng Wang, Hao Chen, Minxiao Wang, Xin Zhang, Chaolun Li

**Affiliations:** 1 Center of Deep-Sea Research, Institute of Oceanology, Chinese Academy of Sciences, Qingdao, China; 2 Laboratory for Marine Biology and Biotechnology, Qingdao Marine Science and Technology Centre, Laoshan Laboratory, Qingdao, China; 3 National Marine Environmental Monitoring Centre, Dalian, China; 4 Key Laboratory of Marine Geology and Environment, Institute of Oceanology, Chinese Academy of Sciences, Qingdao, P. R. China; 5 South China Sea Institute of Oceanology, Chinese Academy of Science, Guangzhou, China; 6 University of Chinese Academy of Science, Beijing, China; Academia Sinica, TAIWAN

## Abstract

The alvinellid worm *Paralvinella hessleri* is the only animal that colonizes the hottest part of deep-sea hydrothermal vents in the west pacific. We found *P. hessleri* accumulates exceptionally high level of toxic element arsenic (>1% of wet weight) and tolerated elevated concentrations hydrogen sulphide. Using advanced microscopy, elementary analysis, and genomics and proteomics approaches, we identified a previously unrecognized arsenic-sulfide biomineralization process in *P. hessleri*. Our data suggest that arsenic accumulates within epithelial cell granules, where it likely reacts with sulphide diffused inward from the hydrothermal vent fluid, resulting in the intracellular formation of orpiment (As₂S₃) minerals. In this “fighting poison with poison” manner, the highly toxic arsenic and sulphide were simultaneously detoxified in the form of orpiment minerals within the intracellular granules of the single layer of epithelial cells. This process represents a remarkable adaptation to extreme chemical environments. Our study provides new insights into understanding animals’ environment adaptation mechanisms and the diversity and plasticity of biomineralization.

## Abstract 2

The deep-sea worm Paralvinella hessleri thrives in extreme hydrothermal vents, tolerating high arsenic and hydrogen sulfide levels. This study shows that it detoxifies both by forming orpiment (As₂S₃) minerals inside epithelial cells; this unique biomineralization reveals a novel adaptation to toxic environments.

## Introduction

While most animals are restricted to habitats with a narrow range of modest conditions, some have adapted to the extreme environments that are hostile, or even lethal to others [1]. These “extreme” animals are fascinating for scientific research because they have evolved unique mechanisms that adapt to the seemingly insurmountable environmental challenges [1,2]. The alvinellids, a family of polychaete worms comprising two genuses, namely, the *Alvinella* and the *Paralvinella*, are one of the most extreme examples of such adaptations [3,4]. The alvinellids are endemic to the deep-sea hydrothermal vents below 1 km water depth where hydrothermal fluids mix with deep-sea waters [5]. Some thermophilic alvinellids, such as the famous “Pompeii worm” *Alvinella pompejana*, even preferably inhabit the areas close to the extrusions of mid to high-temperature vents that no other animals can tolerate [6,7]. This extreme habitat provides both opportunities and challenges for alvinellids. The warm, gas-rich vent fluid promotes high primary productivity [8,9] and deters predators, creating an abundant food supply for the alvinellids. However, the fluctuating physical and chemical conditions, as well as the high levels of sulphide and heavy metals [10], are lethal to most metazoan [11,12]. Among all the environmental stresses, the ability of alvinellids to cope with high concentrations of arsenic is particularly interesting.

Arsenic, which is a highly toxic and carcinogenic metalloid, is a common environmental pollutant causing severe health problems worldwide [13]. Due to its similar chemical properties to phosphorous, arsenic could be taken by transporters of minerals and nutrients through either direct cell diffusion or trophic transfer. The two most abundant valence states of arsenic in aqueous systems are the inorganic species arsenite [As(III)] and arsenate [As(V)], between which arsenite is about 10 times more toxic than arsenate [14,15]. Although seawater contains low levels of arsenic (approximately 10–20 nM) [16], hydrothermal fluids typically have elevated levels of this toxic element. Moreover, the deep-sea hydrothermal ecosystem lacks photosynthetic phytoplankton and macroalgae, which could convert toxic inorganic arsenic species to non-toxic arsenobetaine [17,18]. As a result, alvinellids are primarily exposed to toxic inorganic arenite and arsenate [19,20]. Recent studies have shown that the East Pacific Rise (EPR) alvinellid *A. pompejana* [19] and the Juan de Fuca and Explorer ridges alvinellids *Paralvinella sulfincola*, as well as *P. pamiformis* which lives in cooler regimes at vents, are all hyperaccumulators of arsenic while the majority are toxic inorganic arsenic [21]. However, how and why the alvinellids accumulate and cope with such a high concentration of arsenic is still unknown [22,23].

*Paralvinella hessleri*, a bright yellow colored alvinellid worm, is one of the dominant species in the West-Pacific Back-Arc hydrothermal systems, such as the hydrothermal fields of Izu-Ogasawara Arc and the Okinawa Trough [6,24]. Like the Pompeii worm *A. pompejana*, *P. hessleri* preferably inhabits niches close to warm and highly toxic hydrothermal venting [25]. Compared with EPR, these Back-arc hydrothermal vents emit a much higher concentration of arsenic [20], making the habitats even more challenging for *P. hessleri*. Our environmental chemistry survey showed that *P. hessleri* bio-accumulated and tolerated a staggeringly high level of arsenic (approximately 10,000 μg/g, whole body, fresh weight), which is about one magnitude higher than most known arsenic hyperaccumulators (such as polychaete worm *Arenicola marina* and brake fern *Pteris vittata* accumulate inorganic arsenic, and polychaete worm *Tharyx marioni* accumulate organic arsenic) [26–28]. During our light and electron microscopy analysis, we unexpectedly found that the intracellular granules in the *P. hessleri*’s epithelial cells, which give the worm’s unique bright yellow color, are comprised of biomineralized arsenic minerals. Through further comprehensive analysis involving micro-Raman microscopy, genomics, and proteomics, our study unveiled a unique “fighting poison with poison” adaptation in *P. hessleri*. This involves the utilization of hydrogen sulphide to detoxify arsenic, which may enable the worm to thrive in the extreme environment of deep-sea hydrothermal vents.

## Results

### Intracellular yellow granules of *Paralvinella hessleri*

**Fig 1:**
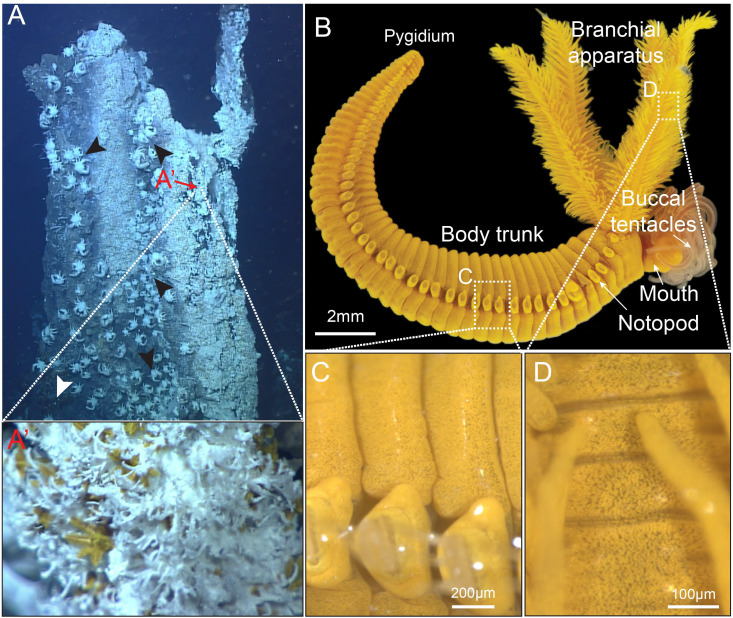
Images of the alvinellid worm, *Paralvinella hessleri.* **A:** A *P. hessleri* colonized hydrothermal vent in Iheya north hydrothermal field. The vent fauna showed apparent variation along the environmental gradients. The areas close to hydrothermal venting were covered with white mucus matt (*P. hessleri* colonies). The squad lobsters *Shinkaia crosnieri* occupied the areas surrounding the *P. hessleri* colonies (indicated by black arrowheads). *Bathymodiolinae* mussels stayed further away (indicated by white arrowhead); **A’:** Close-up image of *P. hessleri* worms close to the hydrothermal venting. **B:** A *P. hessleri* specimen with buccal tentacles extroverted, lateral view. Note that the animal has a bright yellow color; **C:** A close-up image of the notopod; **D:** A close-up image of the stem of branchial apparatus. Both panels **C and D** demonstrate that there are yellow granules in the epidermis of *P. hessleri*.

**Fig 2:**
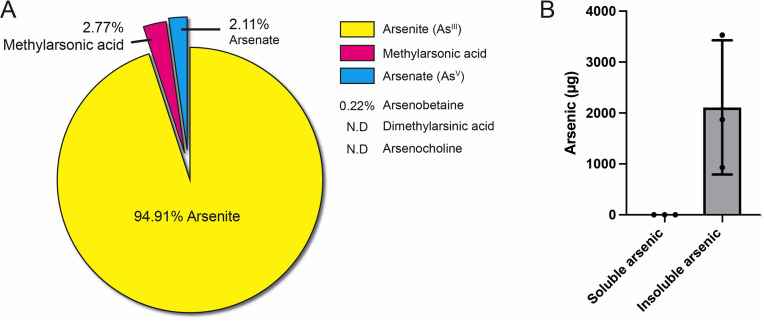
Arsenic speciation and distribution between soluble and insoluble forms in *P. hessleri.* **A:** Percentage of each arsenic species present in *P. hessleri*. **B:** soluble and insoluble arsenic in *P. hessleri* (*n* = 3). The data underlying Fig 2B can be found in S1 Data.

In Iheya North hydrothermal vent fields, the fauna associated with *Paralvinella hessleri* colonized vents showed apparent variation along steep environmental gradients [22]. *P. hessleri* forms monospecific assemblages on chimney walls around vent emissions (Fig 1A and S1 Video). In contrast, other typical vent-associated invertebrates, such as the squat lobster (*Shinkaia crosnieri*) and the bathymodioline mussels (*Bathymodiolus platifrons*), occupy the niches further away from the venting (Fig 1A). Environmental chemistry survey showed that the *P. hessleri* adapted into a harsh environment with a high concentration of toxic compounds. The microbial mat, which *P. hessleri* lives and gazes on, has a high concentration of heavy metals (S1 Fig). Meanwhile, Water chemistry analysis showed that the venting fluid contains a high level of sulphide, with the recorded concentrations over 1 mM [29]. We examined the total arsenic in *P. hessleri* and found the total arsenic of *P. hessleri* worm reaches 10,189 ± 2231.1 μg/g (*n* = 3, fresh weight), estimated almost 1% of the worm’s weight. We then analyzed the species composition of the arsenic present in the worm. The analysis revealed that the primary arsenic species were inorganic species (Fig 2A), specifically arenite (As^III^), which accounted for 92.21% of the total arsenic. In addition, vast majority of the arsenic in *P. hessleri* are insoluble form incorporated within worm’s tissue (Fig 2B). These findings indicate that *P. hessleri* has the capacity to bioaccumulate and, more importantly, cope with large quantities of arsenic in its toxic inorganic form.

**Fig 3:**
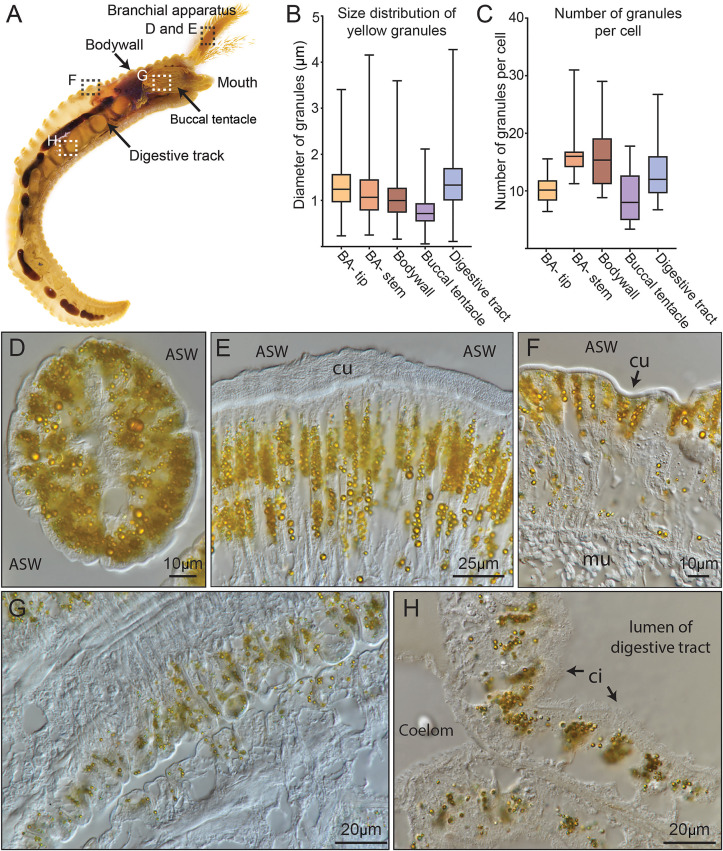
Microscopy analysis of the yellow granules. **A:** A longitudinally sectioned paraffin-embedded *P. hessleri* specimen, demonstrating the internal structure of the *P. hessleri* worm. **B:** Size distribution of the yellow granules in the major tissues of *P. hessleri*. **C:** Number of yellow granules per cell in the major tissues of *P. hessleri*. **D:** Cross section of branchial apparatus tip. **E:** Longitudinal section of *P. hessleri* branchial apparatus stem. **F:** Longitudinal section of *P. hessleri* body wall. **G:** Longitudinal section of *P. hessleri* buccal tentacles. **H:** Cross section of *P. hessleri* digestive tract. ASW: ambient seawater. ci: cilia. cu: cuticle. The data underlying Fig 3B and 3C can be found in S1 Data.

The bright yellow color observed in *P. hessleri* appears to emanate from yellow pigments present in its epidermal tissues (Fig 1B, 1C, and 1D). This raises questions, as denizens of deep-sea chemosynthetic ecosystems, accustomed to total darkness, typically exhibit subdued coloration, being either greyish white (in the absence of pigments, as exemplified by the squat lobster) or adorned in hues of orange to dark red (in the case of hemoglobin-containing organisms such as the giant tubeworm *Riftia pachyptila*). We then transverse sectioned the *P. hessleri* worm and analyzed the distribution of the yellow granules in its major tissues (Fig 3A, 3B, and 3C). The color of the worm is attributed to spherical granules housed within its epidermis. Within the branchial apparatus, which is the feather-like sensory tissue of the worm [25], epithelial cells at both the tips (Fig 3D) and stem (Fig 3E) harbor a substantial quantity of yellow granules, characterized by average diameters of 1.29 μm and 1.18 μm, respectively (Fig 3B and 3C). Similar yellow granules can also be found in the worm’s body wall epithelial cells (Fig 3F), with an average diameter of 1.04 μm (Fig 3B and 3C). Unexpectedly, these yellow granules are also evident in the buccal tentacles (Fig 3E) and the digestive tract (Fig 3F). The buccal tentacles, which might be involved in gas exchange, features smaller yellow granules compared to those within the branchial apparatus and body wall epidermis, with an average diameter of 0.77 μm (Fig 3B, 3C, and 3G). While in the digestive tract, relatively large granules are discernible in the ciliated goblet-like cells (Fig 3B, 3C, and 3H).

The ubiquity of yellow granules not only in the epidermis but also within internal organ epithelial cells dispels the hypothesis of their role as aposematic signals. Notably, all cells housing these granules maintain direct interface-cells with seawater (Fig 3 and S1 Fig). Furthermore, a spatial arrangement is noted, with the granules congregating in proximity to the seawater interface within these cells. In the branchial apparatus and body wall epidermis, for instance, they are conspicuously localized near the cuticle (S1A, S1B, and S1C Fig). Similarly, within the buccal tentacles, yellow granules aggregate proximal to the seawater-tissue interface (S1D Fig). In the digestive tract, these granules cluster at the apical side of the cells, adjacent to the lumen of the tract (S1E Fig).

### The yellow granules are intracellular sequestration vacuoles

**Fig 4:**
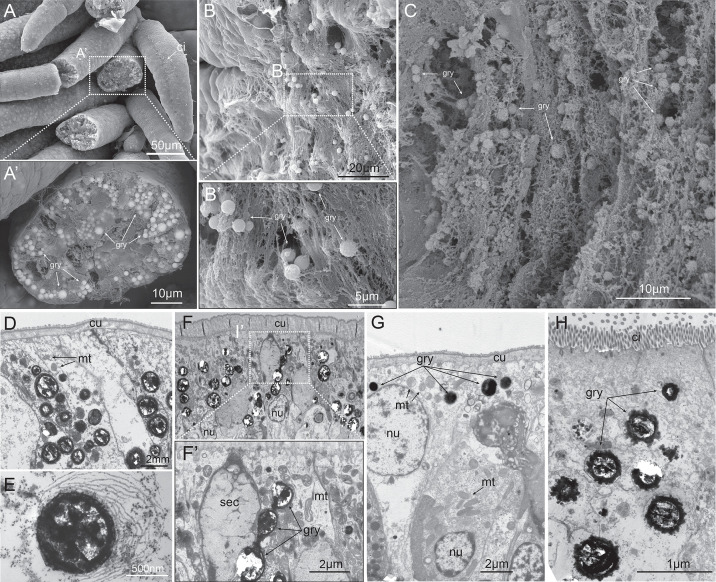
SEM and TEM analysis of the yellow granules. **A:** A SEM image of *P. hessleri* branchial apparatus; **A’:** A magnified region showing the electron-dense intracellular yellow granule. **B:** A SEM image of *P. hessleri* body wall; **B’:** A magnified region showing the electron-dense intracellular yellow granules. **C:** A SEM image of the digestive tract yellow granules. **D:** A TEM image of *P. hessleri* branchial apparatus. **E:** A close-up image of a typical branchial apparatus yellow granule. **F:** A TEM image of *P. hessleri* body wall; **F’:** A magnified region showing yellow granules and a secretion cell. **G:** A TEM image of buccal tentacles yellow granules; **H:** A TEM image of digestive tract yellow granules. ci: cilia; cu: cuticle, mt: mitochondria; gry: yellow granule, nu: cell nuclei.

We then employed electron microscopy ultrastructural analysis to elucidate the detailed morphology of the yellow granules. The scanning electron microscopy (SEM) analysis of the branchial apparatus (Fig 4A), body wall (Fig 4B), and digestive tract (Fig 4C) revealed that the granules with smooth surface are embedded within the cytosol of cells, suggesting the granules are products of biologically controlled process. Notably, under back-scatter detection mode, the granules exhibited enhanced brightness, indicating their high electron density relative to surrounding cellular components.

To rule out the possibility that these yellow granules represent intracellular unidentified symbionts, we used transmission electron microscopy (TEM) to conduct a detailed examination of both the granule-containing cells and the granules themselves. The hypothesis that the yellow granules are intracellular symbionts necessitates the presence of specific host cell mechanisms, such as supportive membranes and lysosomes, to manage the symbiosis. Additionally, the symbiont itself should exhibit distinct subcellular characteristics differentiating it from other organelle type [30–32]. Our TEM results revealed the yellow granules located within the intracellular cytoplasm (Fig 4D–4H), enclosed in vacuoles or liposomes delimited by apparent membrane-like structures (Fig 4E). However, the granules consistently displayed heavy staining, suggestive of a composition rich in heavy metals and lacking recognizable internal structures. Collectively, these observations from both SEM and TEM analyses support the classification of the yellow granules as electron-dense intracellular spherocrystals, rather than intracellular symbiotic bacteria.

#### The yellow granules are comprised of biomineralized orpiment (As_2_S_3_) minerals.

**Fig 5:**
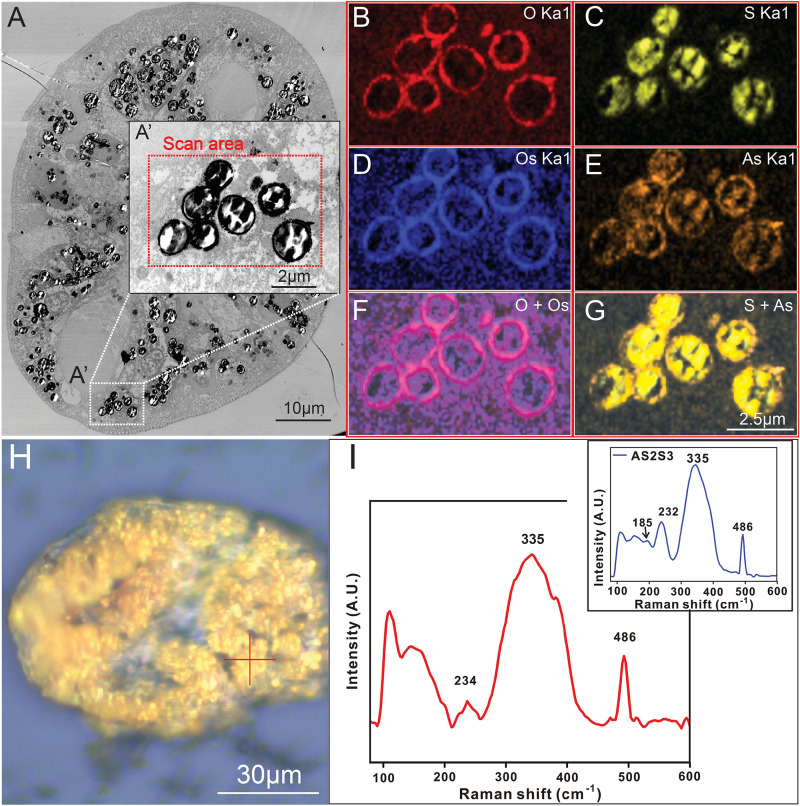
STEM-EDS mapping and micro-Raman spectrometry analysis of the yellow granules. **A:** Bright-field STEM image of yellow granules in the fine tip of branchial apparatus; **A’:** A close up image of the STEM-EDS mapping scanning area; **B–E:** EDS mapping of Oxygen, Sulphur, Osmium, and Arsenic elements of the yellow granules; **F:** Merged image of Oxygen and Osmium EDS mapping; **G:** Merged image of Sulphur and Arsenic EDS-mapping; **H:** Optical image of branchial apparatus yellow granules for micro-Raman analysis; **I:** Raman spectra of yellow granules; Redcross in **H** micro-Raman spectrometry sampling point; Inset in **I:** Raman spectra from pure As_2_S_3_. The data underlying Fig 5I can be found in S2 Data.

To investigate the elemental composition of the yellow granules, we conducted an elemental analysis using STEM-EDX (Scanning Transmission Electron Microscopy with Energy-Dispersive X-ray Spectroscopy) mapping. Within the branchial apparatus (Fig 5A), the EDX mapping revealed that the yellow granules are encased by a thin sheath abundant in oxygen, osmium, and arsenic, as depicted in Fig 5B, 5C, and 5D. In contrast, the internal composition of the granules primarily comprises arsenic and sulphur (Fig 5E, 5F, and 5G). The EDX mapping analysis of the granules in the body wall and digestive tract yielded consistent results (S2 Fig). Consequently, these findings collectively confirm that the yellow granules are intracellular sequestration granules employed for the detoxification of arsenic and sulphur.

Arsenic can form compounds with sulphur, leading to diverse minerals such as orpiment (arsenic trisulfide), realgar (tetra-arsenic tetra-sulphide), dimorphite (tetra-arsenic trisulfide), and arsenic pentasulfide. While realgar and dimorphite minerals display hues of orange-red, orpiment, which is the thermodynamically favored arsenic sulfide phase under reducing, low-temperature, and near-neutral pH conditions, is distinguished by its bright yellow color [33]. To confirm the mineral identity of the yellow granules, a micro-Raman spectrometry analysis was conducted (Fig 5H and S3A Fig). The micro-Raman spectra obtained from the yellow granules (Fig 4I and S3B Fig) revealed arsenic trisulfide at 234, 335, and 486 cm^−1^. Considering several contextual considerations: (1) the reductive nature of the hydrothermal environment; (2) the prevalent presence of As^III^ in the total arsenic composition of *P. hessleri* (Fig 2A); and (3) the distinctive reddish-orange coloration [33] associated with arsenic pentasulfide (realgar) which is different from the observation in *P. hessleri*, the results derived from the Raman spectrometry analysis suggest that the yellow granules are mostly composed of orpiment minerals.

### Yellow granules are inserted with vacuolar membrane

**Fig 6:**
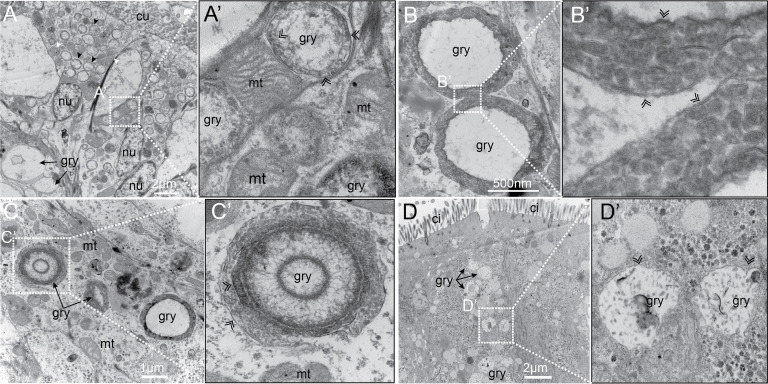
TEM analysis of the NaOH-treated *P. hessleri* tissues. **A:** TEM image of NaOH-treated branchial apparatus; **A’:** A close-up image of yellow granules’ membrane structures and intra-membrane vacuoles; **B:** TEM image of two body wall yellow granules; **B’:** A close-up image showing the two sets of membranes, and numerous vesicles between the membranes; **C:** ci: cilia, cu: cuticle; gry: yellow granule; mt: mitochondria; nu: nuclei; black arrowhead: yellow granule; white arrowhead: mitochondria; double line arrowhead: lipid bilayer; white arrow: protrusions on the gut yellow granule.

The accumulated arsenic in the membrane and yellow granules presented a significant hindrance to the detailed observation of the membrane structure using TEM. Given the solubility of orpiment mineral in alkaline solutions, a 0.1 M NaOH treatment was provided to the *P. hessleri* specimens to dissolve the orpiment. Subsequent TEM analysis of the membrane structure revealed that the yellow granules in branchial apparatus (Fig 6A), body wall (Fig 6B), and buccal tentacles (Fig 6C) were enveloped by two sets of lipid bilayer membranes. Within the intermembrane space, vessels were identified, each encased in a lipid bilayer membrane (Fig 6A–6C). Intriguingly, a comparable structure has recently been reported in sponge-associated bacteria *Entotheonella sp.*, wherein arsenic is mineralized and detoxified within intracellular vacuoles [34]. The yellow granules within digestive track cells have one single layer of membrane structure (Fig 6D), suggesting these yellow granules might be formed through different mechanisms. These findings collectively suggest that the orpiment minerals undergo controlled biological mineralization, and the membrane surrounding the granules serves as the micro-niche responsible for the transportation and concentration of arsenic.

### The proteomic and molecular analysis of orpiment biomineralization

Given the presence of membrane structures within the yellow granules, we hypothesize that these membranes may regulate orpiment mineralization and subsequently serve as deposition layers during the mineralization process. We then conducted whole-genome sequencing, proteomics and molecular analysis to further understand the major proteins that are involved in governing the orpiment biomineralization in the *P. hessleri*. Using PacBio long-read and Illumina short-read sequencing, we assembled the *P. hessleri* genome into 2,446 high-quality contigs (N50 = 639.5 Kbps), with the assembled genome size of 640.3 Mbp*.* The BUSCO (Benchmarking Universal Single-Copy Orthologs) analysis of the *P. hessleri* genome revealed a completeness of 95.7% (with 86.2% Single-Copy BUSCOs, 9.5% Duplicated BUSCOs, 0.8% Fragmented BUSCOs, and 3.5% Missing BUSCOs). Approximately 93.7% of the Illumina short-reads could be successfully mapped to the *P. hessleri* genome, indicating the genome assembly has high completeness (S2 Table). A total of 22,289 protein-coding genes were identified, 99.30% of which (22,128 genes) could be annotated in databases including NR, Swissport, KEGG, InterPro, GO, and Pfam (S4 Fig). The RNA-seq gene expression data covering five major tissues of the *P. hessleri* worm was provided as S3 Table.

**Fig 7:**
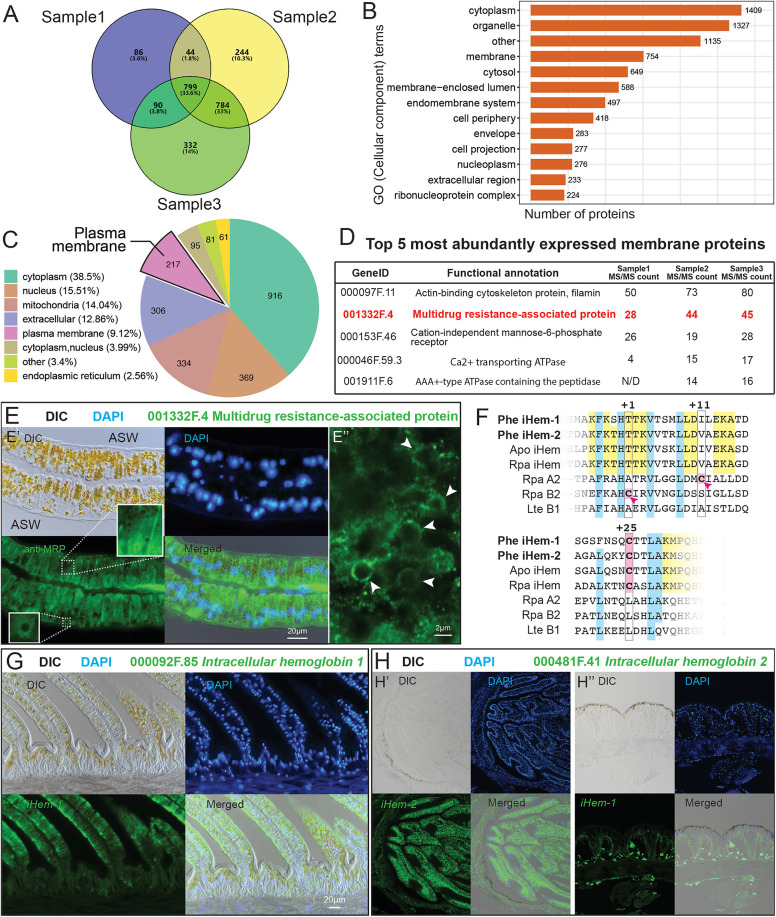
Analysis of the key genes involved in *Paralvinella hessleri* orpiment biomineralization. **A:** Proteins identified in three *P. hessleri* yellow granules samples. **B:** Gene ontology cellular component classification (GO CC) of *P. hessleri* yellow granule proteome. **C:** Subcellular locations analysis of *P. hessleri* yellow granule proteome. **D:** Top 5 most abundantly expressed membrane proteins in the *P. hessleri* yellow granule proteome. **E:** Immuno-histochemistry analysis of the 001332F.4 Multidrug resistance-associated protein. **F:** Sequence alignment analysis of two *P. hessleri* intracellular hemoglobins. **G:** Fluorescent in situ hybridization analysis of gene encoding *P. hessleri* Intracellular hemoglobin 1 (iHem-1, 000092F.85) in the branchial apparatus. **H:** Fluorescent in situ hybridization analysis of gene encoding *P. hessleri* Intracellular hemoglobin 2 (iHem-2, 000481F.41) in the **H’** branchial apparatus and **H”** body wall of the *P. hessleri*.

Using the *P. hessleri* genome sequences as a reference, we performed a proteomic analysis to characterize the protein composition of the yellow granules. Yellow granules were selectively isolated by Percoll gradient centrifugation with subsequent removal of cytoplasmic proteins, followed by a high-sensitivity 4D label-free proteomics profiling of yellow granule’s proteome. From three yellow granule samples, we identified 1,019, 1,871, and 2,005 proteins, which yielded a dataset comprising 2,379 proteins (Fig 7A and S3 Table). Subsequent gene ontology classification analysis unveiled that 1,327 proteins fell under the “GO CC (cellular component) Organelle” category, indicative of successful enrichment of the yellow granules (Fig 7B). Meanwhile, GO analysis also revealed that the proteins associated with subcellular membrane-bounded organelle (Fig 7B), such as small ribosomal subunit (GO:0000314), organellar ribosome (GO:0000313), myelin sheath (GO:0043209) and organelle membrane (GO:0031090) are enriched, which align with the results of TEM observations (Fig 6).

We then focus on the proteins predicted to localize to the plasma membrane which may involve in the transportation of arsenic. Out of the 2,379 yellow granule proteins, 217 were categorized as putative plasma membrane residents (Fig 7C). Interestingly, a Multidrug Resistant-associated Protein (MRP) was found among the most abundant membrane proteins (Fig 7D). The MRP belongs to the superfamily of ATP-binding cassette (ABC) transporters which transport a variety of molecules across the extra or intracellular membranes.

We generated an anti-MRP polyclonal antibody and conducted an IHC (immunohistochemical) analysis to assess the expression patterns of MRP. As shown in Fig 7E, the IHC results revealed the presence of the fluorescent signal of MRP on the membranes of the branchial apparatus epithelial cells, suggesting that MRP plays a role in the transportation of arsenic or other substrates into the epithelial cells. Notably, at the sub-cellular level, intense MRP expression was identified in close proximity to the yellow granules (Fig 7E’). Confocal microscopy revealed distinct localization of MRP on the membrane surrounding the yellow granules (Fig 7E” and S5 Fig), suggesting its involvement in arsenic transportation.

Interestingly, the proteomics data has also uncovered the presence of two intracellular hemoglobins prominently featured among the predominant proteins in the yellow granule proteome (S4 Table). Alvinellid worms have a dual hemoglobin system, encompassing both extracellular and intracellular variants [35,36]. Analogous to the hydrothermal vent vestimentiferan tubeworms, the extracellular (vascular) hemoglobin in alvinellids has a hexagonal bi-layer (HBL) structure, constituted by multiple globin chains linked by linker chains [37,38]. In contrast, the intracellular hemoglobin is monomeric globin [36]. Sequence alignment analysis demonstrates that both intracellular hemoglobins, namely iHem-1 (000092F.85) and iHem-2 (000481F.41), a +25-cysteine residue conserved among polychaetes inhabiting high-sulphide hydrothermal vent environments but lack +1 or +11 cysteine residues observed in symbiotic vestimentiferan tubeworms (Fig 7F).

We then analyzed the gene expression patterns of both intracellular hemoglobin and genes encoding extracellular hemoglobin (000362F.2) and linker proteins (0007545F.7) with mRNA in situ hybridization. Our FISH results show that genes encoding the extracellular hemoglobin subunits, linker protein are specifically expressed in an internal secretory gland within the worm’s coelom (S6 Fig), which aligns with the transcriptomic RNA-seq results (S7 Fig).

The *iHem-1* is dominantly expressed in *P. hessleri*’s branchial apparatus (Figs 7G and S8). Within the feather-like tips of the branchial apparatus, *iHem-1* is notably present in columnar epithelial cells, where a significant portion of orpiment biomineralization occurs. Along the stem of the branchial apparatus, *iHem-1* is explicitly expressed in epithelial cells associated with orpiment biomineralization, with no detectable expression in the underlying muscle beneath the epidermis (Fig 7G).

In contrast, *iHem-2* displays a more widespread expression profile (Figs 7H and S9). Within the branchial apparatus, *iHem-2* is expressed in the muscle layer beneath the epidermis (S9B Fig). The *iHem-2* are highly expressed in the worm’s buccal tentacles (Fig 7H’) and hemocytes (S9C Fig), which may both be involved gas-exchanging. Additionally, *iHem-2* is expressed in body wall cells (Fig 7H”) and the digestive tract (S9D Fig) of the *P. hessleri* worm, both sites of orpiment biomineralization. Collectively, the proteomics data and expression patterns of both intracellular hemoglobins suggest their involvement in sulphide transport for orpiment biomineralization.

### The “Fighting poison with poison” adaptation

**Fig 8:**
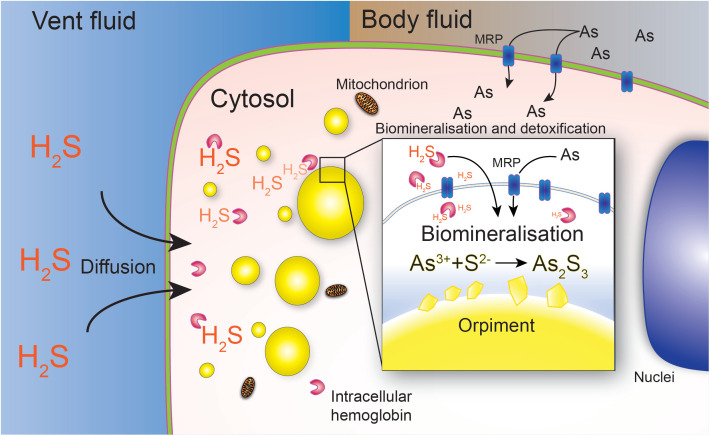
The “Fighting poison with poison” arsenic detoxification in *Paralvinella hessleri.*

We therefore propose a hypothesis asserting that the biomineralization of orpiment in *P. hessleri* represents a distinctive detoxification and environmental adaptation mechanism (Fig 8). *P. hessleri* encounters elevated concentrations of arsenic, which enters the worm through either direct absorption or gazing of the microbial mat. Consequently, *P. hessleri* bioaccumulates arsenic, which is translocated into intracellular vacuoles through transmembrane transporters, such as the ABC transporter Multidrug Resistant-associated Protein. Meanwhile, in the vicinity of hydrothermal venting sites, *P. hessleri* is consistently exposed to high levels of dissolved hydrogen sulphide emanating from vent fluids, which poses a significant threat to metazoans reliant on aerobic respiration [39]. The permeability of H_2_S [40] allows it to freely diffuse into the epithelial cells of *P. hessleri*. Within the epithelial cells, H_2_S forms initial associations with intracellular hemoglobin, which facilitate its transport into intracellular vacuoles. Within the intracellular vacuoles, arsenic reacts with sulphide and deposit insoluble orpiment minerals. This distinctive strategy, akin to a “fighting poison with poison” approach, results in the neutralization of arsenic and H_2_S through their conversion into nontoxic orpiment minerals.

**Fig 9:**
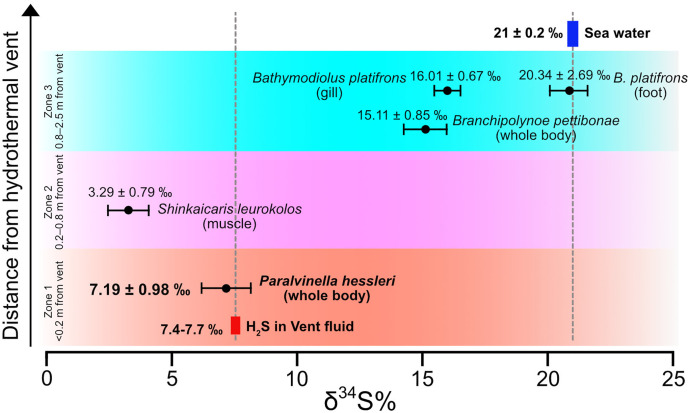
δ^34^S% analysis of the four typical animals collected from hydrothermal vent chimneys. The animals were aligned based on the habitat distance (habitat zones) from the hydrothermal venting. The *Paralvinella hessleri* which lives close to the hydrothermal venting is on the bottom while the *Bathymodiolus platifrons* which lives further away is at the top. The seawater and hydrothermal venting H_2_S δ^34^S% are based on Seal [81] and Gamo [42].

In accordance with this hypothesis, the sulphur elements within the *P. hessleri* worm are obtained through two pathways: (1) sulphur is bioaccumulated within *P. hessleri*’s through biotic metabolism; or (2) sulphur is abiotically fixed through orpiment biomineralization. Given that biotic metabolism, such as bacterial redox metabolism and animal biotic metabolism, exhibit a preference for the accumulation of light isotopes, the origin of sulphur could be discerned through the analysis of the ^34^S/^32^S isotope ratio [41]. Subsequently, we conducted an analysis of the δ^34^S% in *P. hessleri*, as well as four representative vent invertebrates (Fig 9). These included the symbiotic mussel *Bathymodiolus platifrons*, which resides near the periphery of hydrothermal vents; the parasitic polychaete *Branchipolynoe pettibonae*, found within the shell cavity of *B. platifrons*; and the alvinocaridid shrimp *Shinkaicaris leurokolos*, which shares a similar habitat with *P. hessleri*, close to the hydrothermal vents.

The deep-sea symbiotic bathymodioline mussel, *B. platifrons*, inhabiting the periphery region of hydrothermal vents (Zone 3, 0.8−2.5 m form the venting) [22], exhibits a δ^34^S% value of 20.34 ± 2.69‰ in the foot tissue and 16.01 ± 0.67‰ in the gill tissue (Fig 9). The δ^34^S% value in the foot tissue, which closely approximates the δ^34^S% value of sulphur in ambient seawater (21 ± 0.2‰), suggests that the majority sulphur constituents within *B. platifrons* originate from the ambient seawater. Conversely, the δ^34^S% value in the mussel’s gill tissue, which harbors intracellular endosymbionts, reflects the assimilation of lighter sulphur isotopes through the metabolic processes of these endosymbionts. The parasitic polychaete *B. pettibonae*, exhibits a δ^34^S% value of 15.11 ± 0.85‰, approximating to that of the mussel’s gill (Fig 9). Muscle samples from the alvinocaridid shrimp *S. leurokolos*, cohabitating with *P. hessleri* in the Zone 2 of the hydrothermal vent (0.2−0.8 m from the venting) [22], have δ^34^S‰ values of 3.29 ± 0.79 (*n* = 10) (Fig 9). The notably lower δ^34^S‰ value observed in *S. leurokolos* suggests that it primarily obtains its sulphur from chemosynthetic microbes associated with hydrothermal vents.

The δ^34^S‰ value of *P. hessleri* is measured at 7.19 ± 0.98 (*n* = 10), which closely corresponds with the δ^34^S‰ range (7.4–7.7‰) of H_2_S in the Iheya north vent fluid [42] (Fig 9). This alignment in δ34S values between *P. hessleri* and vent H_2_S suggests that *P. hessleri* primarily obtains its sulphur directly from vent H_2_S gas, providing support for the “fighting poison with poison” hypothesis.

## Discussion

The uncovered orpiment biomineralization in this study offers novel insights into understanding the diversity and versatility of animals’ environmental adaptation mechanisms. Particularly intriguing is the utilization of two highly toxic environmental toxins, arsenic and hydrogen sulphide, by *P. hessleri* to neutralize each other in an unexpected “fighting poison with poison” manner.

Arsenic, particularly in its inorganic forms, is recognized as a hazardous environmental pollutant and poses severe global health risks [13,43]. While the bioaccumulation of less toxic organic arsenic forms has been commonly found in terrestrial and shallow water aquatic animals [44,45], the accumulation and tolerance of substantial quantities of inorganic arsenic have been observed primarily within species inhabiting deep-sea hydrothermal vent ecosystems [19–21]. Notably, previous studies have indicated that various alvinellid species, such as *A. pompejana* dwelling in the East Pacific Rise (EPR), and *P. sulfincola* as well as *P. palmiformis* from the Juan de Fuca, and Explorer ridges—regions where vent fluid arsenic concentrations are lower than those encountered by *P. hessleri* exhibit significant levels of arsenic accumulation [19,21]. Interestingly, this substantial arsenic accumulation has been observed not only in thermophilic species but also in *P. palmiformis*, which inhabits cooler vent environments [21]. In all cases, arsenic occurs predominantly in the branchial apparatus and body wall, tissues that coincide with the primary sites of orpiment biomineralization in *P. hessleri*. Although no other alvinellid species documented to date matches the extreme levels observed in *P. hessleri*, these findings suggest that the “fighting poison with poison” strategy may be a conserved adaptation among alvinellids, modulated by the local geochemical conditions of their hydrothermal habitats.

Additionally, it has been reported that several mollusc species from the Pacific Manus Basin (PACMANUS), such as the gastropods *Alvinconcha hessleri* and *Ifremeria nautilei*, as well as the mussel *Bathymodiolus manusensis*, bioaccumulate substantial amounts of inorganic arsenic [20]. In *A. hessleri* and *I. nautilei*, which inhabit niches proximal to hydrothermal venting, arsenic is prominently accumulated in the gills of the gastropods, which are the gas-exchanging organs of these molluscs. This correlation of arsenic bioaccumulation within the sulphide-rich seawater–tissue interface suggests a similar mechanism as observed in *P. hessleri*. These results imply that arsenic, despite being classified as a “nonessential” toxic metalloid [46,47], may play a pivotal role in facilitating adaptation to arsenic and sulphide-rich extreme environments.

The yellow granules observed within **P. hessleri*’s* epithelial cells, which are the site of arsenic detoxification, appear to be the key to this adaptation. We hypothesized two plausible pathways for the production or composition of these granules: either through heretofore undiscovered intracellular symbionts [48–50], or as intracellular detoxification vacuoles [19,51,52]. Prior investigations support the latter hypothesis, indicating the capacity of hydrothermal vent organisms to regulate intracellular trace metals through sequestration into electron-dense vacuoles, potentially facilitating excretion or reducing biological reactivity [10,47,53,54]. Our ultrastructural analysis using SEM and TEM revealed that these yellow granules are situated within the intracellular cytoplasm, enclosed by membrane-like structures, but lack recognizable internal structures typical of symbionts. This evidence collectively supports their classification as electron-dense intracellular organelles, consistent with detoxification vacuoles [55].

To achieve orpiment mineralization, *P. hessleri* needs to enrich both arsenic and sulphide within these biomineralization micro-niches. The STEM-EDS mapping analysis revealed a sheath around the yellow granules rich in oxygen, osmium, and arsenic, but not sulphur. The presence of oxygen and osmium, likely due to the fixation process [56], suggests the encapsulation of the granules within a membranous vascular structure. The presence of arsenic in these membrane structures suggests that transporters of arsenic exist on the membrane. Proteomics analysis of the yellow granules identified a Multidrug Resistant-associated Protein (MRP) among the most abundant membrane proteins (S4 Table). The MRP belongs to the ABC transporter superfamily, known to transport a variety of molecules across membranes. It has been demonstrated that the MRP is an evolutionarily conserved arsenic transporter that is directly associated with a cell’s arsenic resistance. In plants, such as the rice [57] and arsenic super accumulator fern *Peteris vittata* [58], MRPs transport arsenic into intracellular vacuoles [59], which have several arsenic resistance and bioaccumulation mechanisms. Immunofluorescence staining confirmed the presence of MRP on the membranes of branchial apparatus epithelial cells, with intense expression near and on the membrane surrounding the yellow granules, further suggesting its involvement in arsenic transportation into these granules.

The utilization of hydrothermal vent emitted hydrogen sulphide for intracellular orpiment biomineralization may also represent an adaptation strategy of animals’ adaptation to high-sulphide extreme environment. Hydrogen sulphide was abundant in the ancient, anoxic oceans of the Proterozoic, where it served as an energy source for early forms of life [60]. Later, the rise of oxygen concentration since the later Proterozoic eliminated H_2_S from most environments led to the rapid diversification of aerobic multicellular organisms and shaped diversification of life on Earth [61]. Today, naturally sustained and high concentrations of H_2_S are only found in some aquatic environments due to localized production through geochemical and biological processes [2,62,63]. Up to date, a few H_2_S detoxification mechanisms have been reported in the animals adapted into high sulphide environments, such as sulphide insensitive COX gene, switch to anaerobic respiration, and avoidance behaviors [64–66]. In deep-sea hydrothermal vent ecosystem, the Vestimentiferan tubeworm could transport H_2_S with extracellular hemoglobins that could reversibly bind to hydrogen sulfide to its symbiotic organ trophosome where sulphide could be detoxified by sulphide-oxidizing endosymbionts [67,68].

The colonies of Alvinellid worms inhabit niches much closer to hydrothermal vents than Vestimentiferan tubeworms. Previous studies have indicated that Alvinellids thrive in an environment where concentrations of H_2_S ranging from 1 to 3 mM are possible, with 100 to 300 µM being commonly observed [3]. The *P. hessleri* is regularly exposed branchial apparatus to sulphide-rich hydrothermal venting fluid, sulphide could penetrate its epidermis, and also invade the worm through buccal tentacles and the gut during respiration and food digestion [3,69].

The *P. hessleri* genome encodes both extracellular and intracellular hemoglobins. Notably, the genes encoding extracellular hemoglobin chains and their linker proteins are specifically expressed in an internal secretory organ that runs longitudinally along the digestive tract (S6 Fig). This expression pattern suggests that these components are secreted into the worm’s coelomic fluid, where they assemble into large extracellular hemoglobin complexes that are too massive to form intracellularly [70–72]. It also implies that extracellular hemoglobins primarily function within the coelomic fluid [73], and are unlikely to be directly involved in orpiment biomineralization occurring within epithelial cells.

In contrast, the four intracellular hemoglobins of *P. hessleri* exhibit distinct tissue-specific expression patterns. While 000481F.41 and 001382F.11 are predominantly expressed in hemocytes, iHem1 (expressed specifically in the branchial apparatus, as confirmed by RNA-seq) and iHem2 are actively transcribed in tissues associated with biomineralization. This finding is further supported by our in situ hybridization and proteomic analyses, indicating that iHem1 and iHem2 may play a role in orpiment formation. Although iHem1 and iHem2 lack the free cysteine residues at the +1 and +11 positions which are thought to mediate reversible sulfide binding in extracellular hemoglobins [36,38,74], they retain a conserved free cysteine at position +25—similar to intracellular hemoglobins of other alvinellids, such as *Alvinella pompejana*. While the +25 cysteine may not function identically to the +1/+11 cysteines, we speculate it could still contribute to sulfide binding. Thus, we propose that orpiment biomineralization in *P. hessleri* may also serve as a detoxification mechanism for intracellular sulfide: intracellular hemoglobins buffer sulfide within epithelial cells, facilitating its subsequent solidification and detoxification through orpiment formation. This strategy would enable *P. hessleri* to manage intracellular sulphide stress independently, without reliance on symbiotic relationships.

Furthermore, this “fighting poison with poison” mechanism uncovered in this study offers compelling insights into the intricacies of animal biomineralization. Biomineralization stands out as a pivotal evolutionary innovation in the broader context of life’s history and has garnered considerable attention in scientific research [75]. Traditionally, our understanding of animal biomineralization has centered on the biologically controlled production of minerals within specific tissues, contributing to their subsequent reinforcement or stiffening [76]. However, the biomineralization of orpiment observed across multiple tissues in *P. hessleri* deviates from this conventional paradigm, as it serves a detoxification function rather than reinforcing these tissues. To date, only two animals, *P. hessleri* and the scaly-foot snail *Chrysomallon squamiferum* [77,78], both inhabiting the H_2_S-rich deep-sea hydrothermal vent ecosystem, are known to process sulphide minerals. Notably, these two species acquire sulphide through distinct mechanisms: *P. hessleri* directly from venting fluid and the scaly-foot snail via sulphide produced by endosymbionts [79]. Despite these differences in sulphide acquisition, the convergent purpose of their biomineralization, namely protecting the animals from toxins, underscores the significance of this mechanism in the context of evolutionary adaptation. We anticipate that our findings will prompt a revaluation of the current understanding of metal and elements usage in marine invertebrates.

In this study, we recognize certain limitations pertaining to our comprehension of *P. hessleri*’s orpiment biomineralization process. Up to date, obtaining *P. hessleri* samples via deep-sea expeditions and ROV dives remains challenging and resource-intensive. The prolonged cultivation of *P. hessleri* in laboratory settings is currently unattainable, and tools for gene loss-of-function studies have yet to be developed for this organism. One aspect pertains to the incomplete comprehension of the arsenic transport mechanism within this organism. While the Multidrug Resistant Associated Protein has been identified, several other potential arsenic transmembrane transporters revealed in the proteomic analysis of yellow granules remain uncharacterized. Additionally, morphological disparities observed through SEM and TEM analyses between the yellow granules found in *P. hessleri*’s digestive tract and those in other tissues suggest potential variations in the pathways leading to their formation. Despite these limitations, our investigation has contributed to the understanding of orpiment biomineralization by analyzing abundantly expressed proteins.

## Materials and methods

### Animal collection

The specimens of *Paralvinella hessleri* was collected during “2016 WPOS Hydrothermal vent and Cold seep joint cruise” from two hydrothermal vents in the JADE hydrothermal field located at the Okinawa trough (Dive98: 127°04′47.596′′ E, 27°16′15.167′′ N, 1328.3 m depth; and Dive104: 127°04′10.3′′ E, 27°14′56.616′′ N, 1,584 m depth), using the remotely operated vehicle (ROV) “Faxian”. The *P. hessleri* worms and other vent animals were collected by using a suction sampler. Most collected *P. hessleri* worms were alive and active when the ROV was retrieved onboard RV “Kexue”.

The specimens were then treated with a variety of methods according to research purposes. For microscopy analysis, the samples were first relaxed in relaxation buffer (0.37 M MgCl_2_ 1:1 diluted with in situ seawater) and fixed with 4% paraformaldehyde (freshly prepared with in situ seawater) at 4 °C overnight. The samples were then washed 3 times with ice-cold 1× phosphate buffered saline (PBS), dehydrated and stored in 70% Ethanol at −20 °C. For electron microscopy analysis, the worms were fixed in electron microscopy fixative (2.5% Glutaraldehyde, 2% Formaldehyde) and stored in 4 °C until use. For DNA, RNA and stable isotope analysis, the samples were snap-freeze in liquid nitrogen and then stored in liquid nitrogen until use.

### Chemistry analysis of vent fluid, microbial mat, and the *P. hessleri* worm

Vent fluid samples were taken by gas-tight isobaric samplers (150 ml) from the hydrothermal vent. The nozzle of the sample was inserted into the orifice of the hydrothermal venting, and the fluid was sampled once a steady temperature reading was obtained. To minimize the loss of gases and precipitation of minerals, fluids were sampled at the end of the ROV dives. The concentrations of potassium (K), sodium (Na), magnesium (Mg), calcium (Ca), lithium (Li), boron (B) in the fluid samples were measured by inductively coupled plasma atomic emission spectrophotometry (ICP-AES, Thermo Scientific Corp.) after appropriate sample dilution using Milli-Q deionized water. The concentrations of molecular hydrogen (H_2_), carbon dioxide (CO_2_), methane (CH_4_), and ethane (C_2_H_6_) were determined by gas chromatography (GC) with thermal conductivity detection (GC-TCD, Thermo Scientific Corp.) analysis of the extracted gas phase with 10% errors. Hydrogen sulphide was measured by the methylene blue method with an error of ±5%.

The heavy metals in the microbial mat which was collected in Dive98 were analyzed using standard ICP-MS method. In brief, the microbial mat was first frozen dry and grounded into fine power. One gram (dry weight) of the fine powder was placed in PTFE digestion tube (PerkinElmer, USA). Samples were digested using 6 mL of concentrated HNO_3_ and 2 mL of H_2_O_2_ in microwave digestion system (Ethos 1, Milestone S.r.l, Sorisole, Italy). After cooling to room temperature, the digested sample were transferred to 50 mL polypropylene tubes, and diluted to 50 mL using ultrapure deionized water. Reagent blanks were processed simultaneously in triplicate. An Agilent 7500cx (Santa Clara, CA, US) inductively coupled plasma mass spectrometer (ICP-MS) was used for the heavy metal analysis. The sample were analyzed in triplicates to quantify the metals.

To assess the distribution of soluble and insoluble arsenic in *P. hessleri*, snap-frozen worms were first weighed and then thoroughly homogenized in 8 mL of filtered seawater. The homogenates were incubated on ice for 1 hr to ensure complete extraction of soluble components, followed by centrifugation at 12,000 × *g* for 30 min at 4 °C. The arsenic concentrations in both the supernatant (soluble arsenic) and the pellet (insoluble arsenic) were then measured using an iCAP TQ ICP-MS system (Thermo Scientific).

### Microscopy analysis

The *P. hessleri* specimens were paraffin embedded according to standard protocol. The samples were first dehydrated by incubating in 100% Ethanol (twice), 100% Ethanol 1:1 Xylene (once), and Xylene (twice), for one hour each at room temperature. The dehydrated samples were incubated in Paraplast plus (Sigma–Aldrich) for 2 hr at 60 °C, and then cooled down to room temperature. Thin sections of 5 μm were cut on a microtome (RM2016, Leica). The sections were dewaxed and rehydrated in Xylene and a graded series of 100%, 95%, 80%, 70% ethanol solutions. The sections were then washed with PBS and mounted with 50% glycerol. The internal structure of the *P. hessleri* images were taken with a Zeiss Discovery V.20 dissection microscope with AxioCam ICc5 camera. The yellow granule images were taken with a Nikon Eclipse Ni microscope with DS-Ri2 camera.

### SEM and TEM

For SEM (scanning electron microscope) analysis, the internal organs of the worm were revealed by gently tear the worm apart by using fine tip tweezers under dissection microscope. The samples were dehydrated in a graded ethanol series, and then critical point dried. The sample were then coated with gold (Sputter/Carbon Thread, EM ACE200) and observed under SEM (VEGA3, TESCAN).

For TEM (transmission electron microscope) analysis, the samples were rinsed with double distilled water, post fixed with 1% osmium tetroxide, then washed with double distilled water. The samples were then rinsed, dehydrated and embedded in Ep812 resin. Ultrathin sections were obtained with an ultramicrotome (thickness 70 nm, Reichert-Jung ULTRACUT E). The sections were then double stained with lead-citrate and uranyl acetate. The sections were observed under the TEM (JEM1200, JEOL) operated under 100 KV.

To remove the As_2_S_3_ in the yellow granules, the fixed samples were treated with 0.1 M NaOH until the bright yellow color of the worm faded. The samples were washed with double distilled water, then fixed again with electron microscope fixative. The ultrathin sections were then obtained according to the same method describe above.

### STEM-EDS mapping

The thin sections for STEM-EDS mapping were prepared with the same method describe in TEM analysis except the lead-citrate and uranyl acetate double staining was omitted. The STEM-EDS mapping analysis was performed on Zeiss Sigma 500 with Gemini Field-Emission SEM.

### Micro-Raman spectrometry analysis

Raman spectra was performed with confocal Raman micro-spectroscope (alpha 300R+, WITec, Ulm, Germany). The excitation wavelength of 532 nm laser is combined with 50×/0.55 (Zeiss, EC, Epiplan-Neofluar, Germany) microscope objective. The scattered light was delivered to the spectrometer via a 50 mm fiber which also acted as a pinhole providing confocally and 20 mW laser power at the sample. Raman spectra were collected using the back-illuminated charge-coupled-device (CCD) camera thermoelectrically cooled to −60 °C and a 600 grooves/mm grating (UHTS 300, spectroscopy resolution 3 cm^−1^) was chosen. The Raman spectrum and each Raman image was detected with 1.5 s integration time as well as a spectral range from 0 to 3,000 cm^−1^.

### DNA extraction, genome sequencing, and genome assembly

The detailed methods are provided in Supporting Information.

### Proteomics analysis of the yellow granules

The yellow granules were enriched by percoll gradient centrifugation method described by Distel and colleagues, 1988 [80]. Briefly, three *P. hessleri* worms (three separate individuals as biological replicates) were homogenate in 10 mL ice cold imidazole-buffer saline (IBS, containing 0.49 M NaCl, 0.03 M MgSO_4_, 0.011 M CaCl_2_, 0.003 M KCl, and 0.05 M imidazole, pH 7.1) on ice. The percoll gradients were formed by centrifuging 6 ml percoll mixed with 4 mL of 2.5× IBS for 30 min at 12,000 × *g*, resulting in final IBS concentration of 1×. Then, 4 mL of worm homogenate was layered on top of the gradients, and was then centrifuged for 1 hr at 13,000 × *g* at 4 °C. The dense yellow granules were separated from the cell plasma and collected as pellet. The yellow granules were then washed three times with 1× PBS.

To investigate the molecular components associated with orpiment biomineralization, we conducted a proteomic analysis focused specifically on the yellow granules. These granules, observed by electron microscopy to be surrounded by membrane-like structures, were hypothesized to harbor proteins crucial for the initiation and regulation of mineralization. Our aim was to enrich for proteins tightly associated with the granule matrix while minimizing cytoplasmic and plasma membrane contamination.

To further purify the granules for proteomics, they were treated with lysis buffer (8 M urea, 1% protease inhibitor cocktail) and sonicated on ice. After centrifugation at 12,000 × *g* for 10 min, the supernatant containing residual soluble proteins was discarded. The remaining undissolved material, enriched for granules, was then dissolved in 0.1 M NaOH. Proteins were extracted by adding a buffer containing 1% SDS and 1% protease inhibitor cocktail. Disulfide bonds were reduced with 5 mM dithiothreitol at 56 °C for 30 min and alkylated with 11 mM iodoacetamide at room temperature in the dark for 15 min.

Mass spectrometry was performed using a Q Exactive^TM^ Plus (Thermo) coupled online to the UPLC. The spray voltage was set to 2.0 kV, and full MS scans (m/z 350–1800) were acquired at a resolution of 70,000. Data-dependent MS/MS analysis was conducted with a normalized collision energy of 28 and a resolution of 17,500, with a dynamic exclusion window of 15.0 s. Raw MS/MS data were processed using MaxQuant (v1.6.15.0) with tandem mass spectra searched against the *P. hessleri* protein database. Trypsin/P was specified as the cleavage enzyme (allowing up to two missed cleavages). Mass tolerances were set at 20 ppm for the first search and 5 ppm for the main search, with fragment ion tolerance at 0.02 Da. Carbamidomethylation of cysteine was set as a fixed modification, and methionine oxidation as a variable modification. Protein and peptide identifications were filtered at a false discovery rate (FDR) of <1%, with a minimum peptide score threshold of >40.

Proteins were annotated by Gene Ontology (GO) into biological process, cellular component, and molecular function categories. For enrichment analysis, two-tailed Fisher’s exact tests were performed, considering GO terms with a corrected *p*-value < 0.05 as significant. Subcellular localization predictions were conducted using WoLF PSORT (with “Animal” selected as the organism type).

### Immunofluorescence and immuno-EM staining

Polyclonal antibodies for multidrug resistance-associated protein (MRP) were generated in rabbits using a synthetic peptide (NGPHASNLSGNIDTHEKTP-C, corresponding to amino acids 291–309 of MRP) conjugated to ovalbumin (Abclonal, Wuhan, China) as the immunogen. The rabbits were immunized with the peptide-ovalbumin conjugate on days 1, 14, 35, and 56. Antisera were collected on day 70.

For immunofluorescent analysis, *P. hessleri* sections (5 μm thick) were dewaxed and rehydrated according to standard procedures. The sections were then blocked with Blocking buffer (2% sheep serum and 2% BSA in Phosphate-buffered saline with 0.2% Tween 20) for 1 hr at room temperature. The sections were incubated with the MRP polyclonal antibody diluted 1:100 in blocking buffer overnight at 4 °C. After washing, the sections were incubated with Alexa Fluor 488-conjugated goat anti-rabbit IgG secondary antibody (Invitrogen) diluted 1:2000 in Blocking buffer for 2 hr at room temperature. The sections were then stained with DAPI and mounted for fluorescence microscopy. For control staining, section incubated with Alexa Fluor 488-conjugated goat anti-rabbit IgG secondary antibody, with no obvious signal detected.

For immuno-EM, glutaraldehyde fixed branchial apparatus were dehydrated through a graded ethanol series (70%, 85%, 95%, 100%; 15–20 min each) while gradually lowering the temperature to –20 °C. Dehydrated samples were infiltrated with graded ethanol:LR Gold resin (Ted Pella) mixtures (3:1, 1:1, 1:3) and then transferred into pure resin, with two to three changes. Embedding and polymerization were performed under UV light at –20 °C for 48 h.

Ultrathin sections (70 nm) were cut using an ultramicrotome (Leica EM UC6) and rinsed with 0.1 M phosphate buffer (PB). Sections were blocked with 1% acetylated bovine serum albumin (BSAc) and 0.15% glycine in PB for 30 min, then incubated with 1:10 diluted rabbit MRP polyclonal antibody for 2 hr. After six rinses in PB, sections were labelled with 10 nm gold-conjugated goat anti-rabbit IgG (GAR; Aurion) for 1 hr. Following further rinses, sections were stained with 2% uranyl acetate for 5 min and imaged using a Tecnai Spirit TEM (120 kV, Thermo Fisher Scientific) equipped with an EMSIS VELETA CCD camera.

### In situ hybridization (ISH) analysis

The targeted DNA fragments were amplified with gene specific primer pairs (S6 Table) with *P. hessleri* cDNA as template. The amplified fragments of were ligated into the pMD18-simpleT vector (Takara) and transformed into *E. coli*. Individual colonies were picked up, and their plasmids were sequenced to confirm the inserts. The templates for in vitro mRNA transcription were amplified using T7 forward GSP (sense probe control) or Sp6 reversed GSPs (antisense probe) combined with either forward or reversed gene-specific primer (S6 Table). Labelled probes and control probes were generated using digoxigenin (DIG)-12-UTP (Roche) with Sp6 and T7 RNA polymerase, respectively.

For ISH analysis, the *P. hessleri* sessions (5 μm thick) were dewaxed and rehydrated according to standard procedures. The sections were washed with PBST (PBS, with 0.1% Tween 20) three times for 10 min each and then permeabilized by 2 μg/mL proteinase K (NEB) in PBST for 15 min at RT. Post-digestion fixation was conducted by incubating the sections in 4% PFA in PBST for 30 min at RT. The sections were washed three times with PBST for 15 min each. Pre-hybridization was conducted by incubating the sections in Hybridization Mix (HM, containing 50% formamide, 5× saline–sodium citrate (SSC), 0.1% Tween 20, 10 μg/mL heparin, 500 μg/mL yeast tRNA) for 1 hr at 55 °C. *Then*, the in situ hybridization was performed by incubating the sections in approximately 0.5 ng/μL fluorescein (Roche)-labelled probed prepared in fresh HM overnight at 55 °C. The sections were washed three times with 2× SSC for 15 min each at 55 °C, cooled down to room temperature and washed three times with PBST. The slices were washed with PBST three times and then blocked with blocking buffer for 1 hr at room temperature. incubated with 1:2500 diluted anti-DIG-POD (Roche) for 2 hr at RT and washed with PBST for six times and TNT buffer (10 mM Tris-HCl pH 8.0, 0.15 M NaCl, 0.1% Tween 20) for three times. The gene expression signal was developed using the TSA Plus Fluorescein kit (Akoya Biosciences). Finally, the slices were washed with PBST, stained with DAPI, and mounted with ProLong Diamond Antifade Mountant (Thermo Fisher) for fluorescence microscopy. In situ hybridizations were repeated using three individual worms, yielding consistent results. Control hybridizations were performed with T7-generated sense probes under the same conditions, with no obvious signal detected.

### Stable isotope analysis

In the laboratory, all faunal samples for stable isotope analysis were freeze-dried and homogenized in an agate mortar. One mg tissue was placed in tin capsules for carbon and sulphur isotope analyses. A subset was acidified to remove inorganic carbon and measure the δ^13^C signature of organic carbon only. Acidification was carried out by adding drops of 0.1 M HCl, until effervescence ceased. The sample was then dried at 60 °C under a fume extractor to evaporate the acid. To prevent the loss of dissolved organic matter, samples were not rinsed.

The carbon isotopic compositions were analyzed using an elemental analyzer (Flash EA 1112Ht, Thermo Fisher Scientific, San Diego, CA, USA) coupled with an isotope-ratio mass spectrometer (Finnigan Delta V Advantage, Thermo Fisher Scientific). The sulphur isotopic compositions were analyzed using an elemental analyzer (Flash EA 1112Ht, Thermo Fisher Scientific, San Diego, CA, USA) coupled with an isotope-ratio mass spectrometer (IsoPrime JB144, IsoPrime, UK). Stable isotope ratios are expressed in δ (‰) notation with respect to Pee Dee Belemnite (PDB) for δ^13^C, and V-CDT for δ^34^S:


δX (o/oo) =[(Rsample/Rstandard)−1] ×10 ^ 3

where *X* is either ^13^C or ^34^S, R_sample_ is the ^13^C/^12^C or ^34^S/^32^S isotope ratio in the sample and R_standard_ is the ^13^C/^12^C or ^34^S/^32^S isotope ratio for the standard. An internal standard (glycine) was run for every 12 samples. Measurement precision was 0.1‰ and 0.3 ‰ for δ^13^C and δ^34^S values, respectively.

## Supporting information

S1 Table Assessing heavy metal concentrations within the microbe mat habitat of *Paralvinella hessleri* (fresh weight, mg/kg). (DOCX)

S2 Table Mapping rate of *Paralvinella hessleri* genome. (DOCX)

S3 Table RNA-seq analysis of five major tissues of *Paralvinella hessleri* (TPM, transcripts per million). (CSV)

S4 Table Proteomics analysis of *Paralvinella hessleri* yellow granules. (XLSX)

S5 Table Stats of *Paralvinella hessleri* genome sequencing libraries. (DOCX)

S6 Table Primers *for* in situ hybridization probe synthesis. (DOCX)

S1 Fig Microscopy analysis of the distribution of the yellow granules. **A:** a cross section of the tips of branchial apparatus, showing the yellow granules aggregated at the seawater-tissue interface epidermal cells. **B:** a transverse section of the stem of the branchial apparatus, showing the yellow granules are distributed at the seawater-tissue interface epidermal cells. **C:** a transverse section of the body wall, showing the yellow granules aggregated at the seawater-tissue interface epidermal cells. **D:** a cross section of a buccal tentacle, showing the yellow granules aggregated at the seawater-tissue interface epidermal cells. **E:** a cross section of the digestive tract, showing the yellow granules aggregated at interface of the digestive cells lumen of digestive tract. ASW: ambient sea water. Scale bar = 50 μM.
(TIFF)

S2 Fig STEM-EDS analysis of the body wall and gut yellow granules. **A:** Bright-field STEM image of yellow granules in the body wall; **A’:** A close up image of the STEM-EDS mapping scanning area; **B:** EDS mapping of Oxygen, Sulphur, Osmium, and Arsenic elements of the yellow granules; **C:** Bright-field STEM image of yellow granules in the digestive tract; **A’:** A close up image of the STEM-EDS mapping scanning area; **D:** EDS mapping of Oxygen, Sulphur, Osmium, and Arsenic elements of the yellow granules.
(TIFF)

S3 Fig Mapping and micro-Raman spectrometry. **A:** Optical image of body wall yellow granules for micro-Raman analysis; **B:** Raman spectra of yellow granules; Redcross in **A:** micro-Raman spectrometry sampling point; Inset in **B:** Raman spectra from pure As_2_S_3_. The data underlying S3B Fig can be found in S3 Data.
(TIFF)

S4 Fig Functional annotation of *P. hessleri* genes using multiple public databases. **A:** Venn diagram of the number of genes with functional annotation using multiple public databases. **B:** Histogram showing the percentage of genes with functional annotation in each public database. NR: non-redundant proteins of National Center for Biotechnology Information; UniProtKB/Swiss-Prot database of UniProt consortium; KEGG: Kyoto Encyclopedia of Genes and Genomes database; and InterPro: the InterPro database from EMBL-EBI.
(TIFF)

S5 Fig Subcellular localization of 001332F.4 Multidrug Resistance-Associated Protein in yellow granule membranes. **A:** Double fluorescent immuno-histochemistry analysis of the 001332F.4 Multidrug resistance-associated protein and Concanavalin-A which labels cell plasma membrane. The double line arrow head indicate the membrane structure of the membrane structure of yellow granule. **B:** Immuno-EM analysis of 001332F.4 Multidrug Resistance-Associated Protein. Arrowheads indicate localization of the protein on the apical side of the granule membrane.
(TIFF)

S6 Fig Expression of extracellular hemoglobin B2 chain and linker protein in the secretory gland of *P. hessleri.* **A:** FISH image showing specific expression of the extracellular hemoglobin B2 chain (000362F.2) in a secretory gland within coelom of *P. hessleri*. **B:** FISH image showing co-expression of the linker protein (0007545F.7) in the same gland. **C:** Transmission electron microscopy (TEM) image of the secretory gland.
(TIFF)

S7 Fig Expression patterns of extracellular hemoglobin subunits and linker protein genes in *P. hessleri.* Heatmap showing the mean expression levels between three individuals of genes encoding *P. hessleri* extracellular hemoglobin subunits and the associated linker protein, based on RNA-seq data (TPM, transcripts per million) presented in S3 Table.
(TIFF)

S8 Fig Expression patterns of intracellular hemoglobins genes in *P. hessleri.* The expression levels are based on RNA-seq data presented in S3 Table.
(TIFF)

S9 Fig Fluorescent in situ hybridization analysis of gene encoding *P. hessleri Intracellular hemoglobin 2* (*iHem-2*, 000481F.41). **A:** A longitudinally sectioned paraffin-embedded *P. hessleri* specimen, showing the internal structure of the *P. hessleri* worm. **B:** the FISH image of iHem-2 in the branchial apparatus of *P. hessleri*. iHem-2 is expressed in the muscle cells of branchial apparatus stem. **C:** the FISH image of iHem-2 in the embryos and hemocytes of *P. hessleri*. The iHem-2 is expressed in the hemocytes but not in the embryos. **D:** the FISH image of iHem-2 in the digestive tract of *P. hessleri.*
(TIFF)

S1 Data Supporting information data file. (XLSX)

S2 Data The Raman spectra data underlying Fig 5I. (SPC)

S3 Data The Raman spectra data underlying S3B Fig. (SPC)

S1 Methods Detailed methods for Paralvinella hessleri genome sequencing and assembly. (DOCX)

S1 Video Video showing the sampling hydrothermal vent in JADE hydrothermal vents field. (MOV)

## Abbreviations

**:**
ABC: ATP-binding cassette
CCD: charge-coupled-device
EPR: east pacific rise
HBL: hexagonal bi-layer
ICP-MS: inductively coupled plasma mass spectrometer
IHC: immunohistochemical
MRP: multidrug resistant-associated protein
ROV: remotely operated vehicle
