# Supplementary material for: A deep-sea hydrothermal vent worm detoxifies arsenic and sulfur by intracellular biomineralization of orpiment (As2S3)
Source: PLoS Biol. 2025 Aug 26;23(8):e3003291. doi: 10.1371/journal.pbio.3003291 (PMC12380324; doi:10.1371/journal.pbio.3003291)
Supplement: S1 Table — (DOCX) [file pbio.3003291.s001.docx]

| Supplementary Table S1. Assessing heavy metal concentrations within the microbe mat habitat of *Paralvinella hessleri* (fresh weight, mg/kg) | | | | | | | | | | |
| --- | --- | --- | --- | --- | --- | --- | --- | --- | --- | --- |
| *Sampleid* | **Cr** | **Mn** | **Co** | **Ni** | **Cu** | **Zn** | **As** | **Cd** | **Hg** | **Pb** |
| *Sample1* | **6.200** | **869.900** | **0.149** | **3.364** | **37773.95** | **57042.76** | **7290.691** | **539.866** | **336.376** | **9627.841** |
| *Sample2* | **26.349** | **1818.571** | **0.168** | **14.829** | **9129.431** | **74036.12** | **436.704** | **450.331** | **29.499** | **1032.991** |

Cr: Chromium

Mn: Manganese

Co: Cobalt

Ni: Nikkle

Cu: Copper

Zn: Zinc

As: Arsenic

Cd: Cadmium

Hg: Mercury

Pb: Lead
