# Supplementary material for: A deep-sea hydrothermal vent worm detoxifies arsenic and sulfur by intracellular biomineralization of orpiment (As2S3)
Source: PLoS Biol. 2025 Aug 26;23(8):e3003291. doi: 10.1371/journal.pbio.3003291 (PMC12380324; doi:10.1371/journal.pbio.3003291)
Supplement: S2 Table — (DOCX) [file pbio.3003291.s002.docx]

|  |  | **Percentage** |
| --- | --- | --- |
| **Reads** | **Mapping rate (%)** | 93.67 |
| **Genome** | Average sequencing depth | 61.14 |
|  | Coverage (%) | 99.23 |
|  | Coverage at least 4x (%) | 98.86 |
|  | Coverage at least 10x (%) | 98.20 |

**Supplementary Table S2. Mapping rate of *Paralvinella hessleri* genome**
