# Supplementary material for: A deep-sea hydrothermal vent worm detoxifies arsenic and sulfur by intracellular biomineralization of orpiment (As2S3)
Source: PLoS Biol. 2025 Aug 26;23(8):e3003291. doi: 10.1371/journal.pbio.3003291 (PMC12380324; doi:10.1371/journal.pbio.3003291)
Supplement: S5 Table — (DOCX) [file pbio.3003291.s005.docx]

**Supplementary Table S5. Stats of *Paralvinella hessleri* genome sequencing libraries.**

| **Libraries** | **Insert size** | **Total Data (G)** | **Read length (bp)** | **Sequencing coverage (X)** |
| --- | --- | --- | --- | --- |
| **Illumina library** | 350bp | **92** | **150** | **143.75** |
| **Pacbio library** | **-** | **77** | **-** | **120.3** |
| **Total** | **-** | **169** | **-** | **264.05** |
