# Supplementary material for: A deep-sea hydrothermal vent worm detoxifies arsenic and sulfur by intracellular biomineralization of orpiment (As2S3)
Source: PLoS Biol. 2025 Aug 26;23(8):e3003291. doi: 10.1371/journal.pbio.3003291 (PMC12380324; doi:10.1371/journal.pbio.3003291)
Supplement: S6 Table — (DOCX) [file pbio.3003291.s006.docx]

**Supplementary Table S6. Primers for in situ hybridisation probe synthesis**

| Primer names | Primer sequences | Amplicon size |
| --- | --- | --- |
| 000092F.85-F | GGTATCAAAGACGATATCGCTA | 417bp |
| 000092F.85-R | TCACATCATCGGGCCGAGCTTTGA |  |
| 000092F.85-F-T7 | TAATACGACTCACTATAGGGTATCAAAGACGATATCGCTA |  |
| 000092F.85-R-Sp6 | ATTTAGGTGACACTATAGTCACATCATCGGGCCGAGCTTTGA |  |
| 000481F.41-F | TGGGTCTCGCTGACAACATT | 415bp |
| 000481F.41-R | AGTTGACCTCCGACATTAGAG |  |
| 000481F.41-F-T7 | TAATACGACTCACTATAGTGGGTCTCGCTGACAACATT |  |
| 000481F.41-R-Sp6 | ATTTAGGTGACACTATAGAGTTGACCTCCGACATTAGAG |  |
| 000362F.2-F | TTCCGTTTTGCGGCTTTACTGTCGGCT | 524bp |
| 000362F.2-R | CTAAGCTGATAGGTAGCTGGCTATCT |  |
| 000362F.2-F-T7 | TAATACGACTCACTATAGGGATTCCGTTTTGCGGCTTTACTGTCGGCT |  |
| 000362F.2-R-SP6 | ATTTAGGTGACACTATAGACTAAGCTGATAGGTAGCTGGCTATCT |  |
| 000745F.7-F | TCCTGTGCCTGACCCTCTGG | 739bp |
| 000745F.7-R | TGGTCATCCTTGCCATAGGA |  |
| 000745F.7-F-T7 | TAATACGACTCACTATAGTCCTGTGCCTGACCCTCTGG |  |
| 000745F.7-R-Sp6 | ATTTAGGTGACACTATAGTGGTCATCCTTGCCATAGGA |  |
