# Supplementary figures and images for: A deep-sea hydrothermal vent worm detoxifies arsenic and sulfur by intracellular biomineralization of orpiment (As2S3)
Source: PLoS Biol. 2025 Aug 26;23(8):e3003291. doi: 10.1371/journal.pbio.3003291 (PMC12380324; doi:10.1371/journal.pbio.3003291)

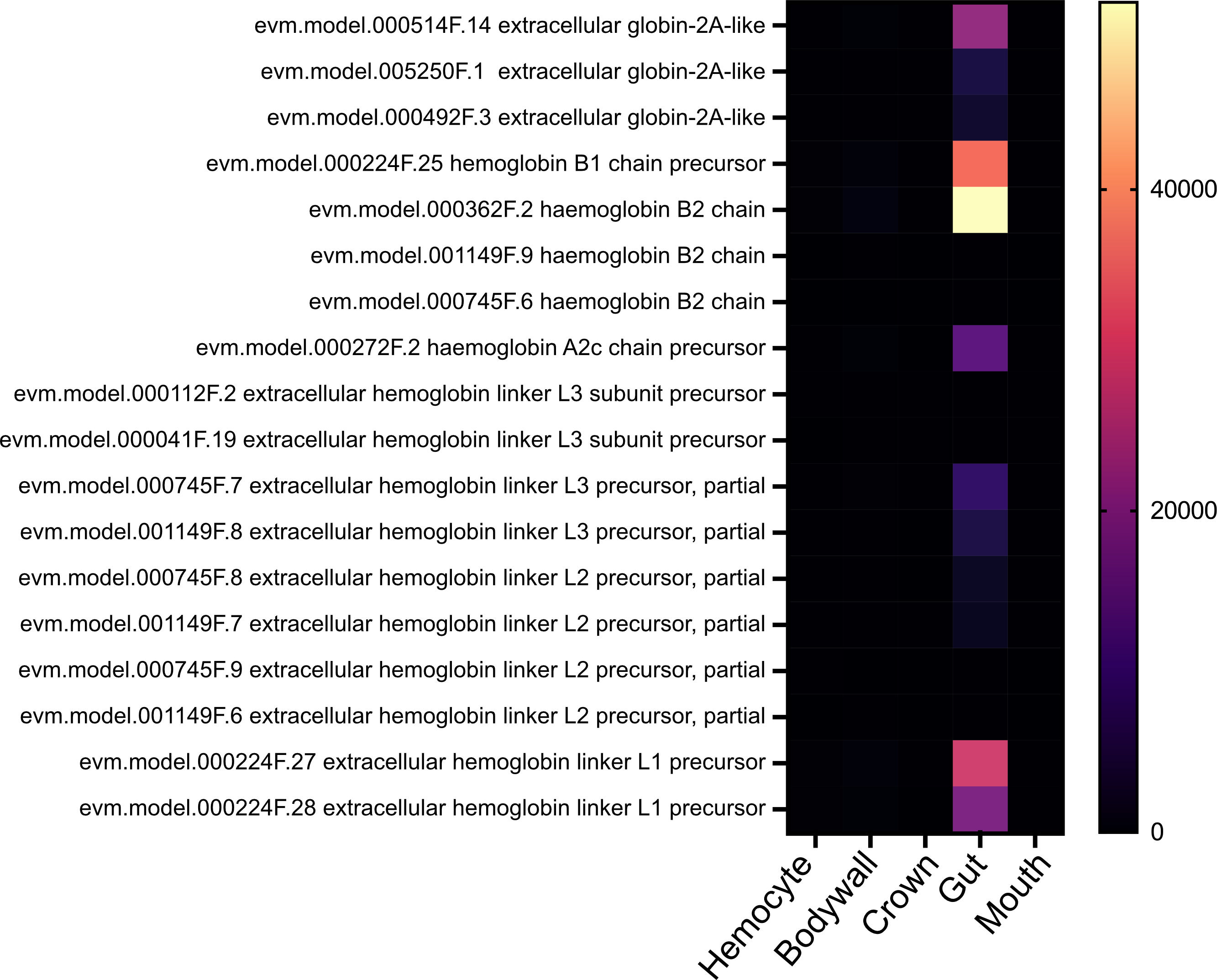

Supplement: S7 Fig — Heatmap showing the mean expression levels between three individuals of genes encoding P. hessleri extracellular hemoglobin subunits and the associated linker protein, based on RNA-seq data (TPM, transcripts per million) presented in S3 Table. (TIFF) [file pbio.3003291.s013.tiff]

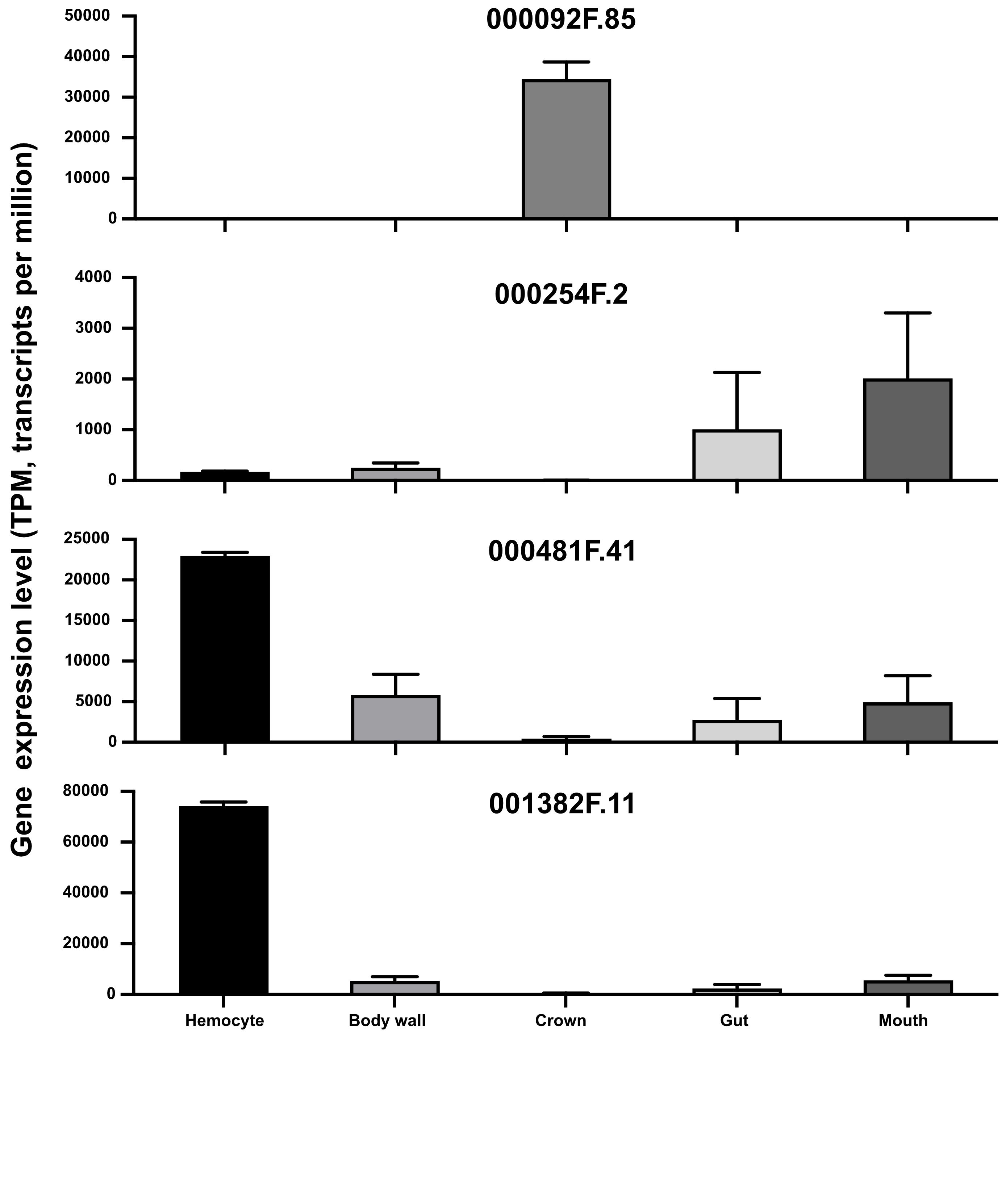

Supplement: S8 Fig — The expression levels are based on RNA-seq data presented in S3 Table. (TIFF) [file pbio.3003291.s014.tiff]
