# Supplementary material for: A deep-sea hydrothermal vent worm detoxifies arsenic and sulfur by intracellular biomineralization of orpiment (As2S3)
Source: PLoS Biol. 2025 Aug 26;23(8):e3003291. doi: 10.1371/journal.pbio.3003291 (PMC12380324; doi:10.1371/journal.pbio.3003291)
Supplement: S1 Methods — (DOCX) [file pbio.3003291.s019.docx]

**DNA extraction, genome sequencing, and genome assembly**

Genomic DNA was extracted from a single *P. hessleri* worm using modified CTAB method. The procedures were as follow: the worm was first dissected, and its gut which might contain DNA contamination from ingested food was carefully removed. Next, the worm was grounded into fine powder using liquid nitrogen and transferred to CTAB buffer (2% CTAB Cetyltrimethylammonium bromide, 100 mM Tris-HCl pH=8.0, 20mM EDTA, 1.4M NaCl, 0.2% β-mercaptoethanol, and 0.1 mg/mL proteinase K). The mixture was incubated at 4ºC for 2 hrs. Genomic DNA was then extracted using the routine phenol/chloroform method. Quality of DNA was checked by PFGE (pulsed field gel electrophoresis). Concentration of DNA was assessed by using the dsDNA HS assay on a Qubit fluorometer v3.0 (Thermo Fisher Scientific).

A total of 1 μg DNA was used to obtain approximate 92 Gb of Illumina Novaseq reads with the paired-end mode and a read length of 150 bp. Meanwhile, a total of 10 μg of genomic DNA was used to obtain approximate 77 Gb of Pacific bioscience RS II long reads (Suppl. Tab. S4). The genome size was estimated based on a k-mer distribution analysis. FALCON^1^ was used for long-read assembly with parameter set as “length _cutoff = 5000; pa_HPCdaligner_option: -k =14, -h = 128, -l = 2000, -w = 8, -T = 8, -s = 700, -M = 32). The completeness of the genome was validated by checking the BUSCO database^2,3^.

**Gene prediction and annotation**

Repeats and transposable elements were annotated using the RepeatModeler 1.0.10 and RepeatMasker 3.3.0 pipeline. The species-specific repeat library was annotated with RepeatModeler. Three approaches: homology-based predictions, de novo pre- dictions, and transcriptome-based predictions were used to predict protein coding genes. For homology-based prediction, protein sequences from seven lophotrochozoan species *Capitella teleta*, *Helobdella robusta*, *Lingula anatine*, *Patinopecten yessoensis*, *Crassostrea virginica*, *Lottia gigantean*, and *Octopus bimaculoides* were aligned to the genome using tblastn.

To optimize the genome annotation, the RNA-Seq reads from which were aligned to genome fasta using Hisat (v2.0.4) / TopHat (v2.0.11) with default parameters to identify exons region and splice positions. The alignment results were then used as input for Stringtie (v1.3.3)/Cufflinks (v2.2.1) with default parameters for genome-based transcript assembly.

The non-redundant reference gene set was generated by merging genes predicted by three methods with EvidenceModeler (EVM, v1.1.1) using PASA (Program to Assemble Spliced Alignment) terminal exon support and including masked transposable elements as input into gene prediction. Individual families of interest were selected for further manual curation by relevant experts.

Gene functions were assigned according to the best match by aligning the protein sequences to the Swiss-Prot using Blastp (with a threshold of E-value ≤ 1e−5). The motifs and domains were annotated using InterProScan70 (v5.31) by searching against publicly available databases, including ProDom, PRINTS, Pfam, SMRT, PANTHER and PROSITE. The Gene Ontology (GO) IDs for each gene were assigned according to the corresponding InterPro entry. We predicted the proteins function by transferring annotations from the closest BLAST hit (E-value <10^-5^) in the Swissprot database and DIAMOND (v0.8.22) /BLAST hit (E-value <10^-5^) hit (E-value <10^-5^) in the NR database. We also mapped gene set to a KEGG pathway and identified the best match for each gene.

**RNA extraction and RNA-seq transcriptomic analysis**

Total RNA from five major tissues of the *P. hessleri* worms (three individuals as biological replicates), including the hemocytes, the body wall, the branchial crown, the gut, and the mouth region, was extracted using TRIzol reagent (Invitrogen) according to manufactory’s protocol. The total RNA was treated with TurboDNA free kit (Ambion) to remove DNA contamination. The RNA samples were sent to Novogene for messenger RNA-seq. The libraries were sequenced on Illumina Hiseq4000 platform to produce pair ends of 150 bp read length. For each dataset, gene expression levels were quantified by directly mapping clean reads to *the P. hessleri* transcripts model using bioinformatics tool Salmon parameters with default parameters.

1 Chin, C. S. *et al.* Phased diploid genome assembly with single-molecule real-time sequencing. *Nat Methods* **13**, 1050-1054 (2016). https://doi.org/10.1038/nmeth.4035

2 Manni, M., Berkeley, M. R., Seppey, M. & Zdobnov, E. M. BUSCO: Assessing Genomic Data Quality and Beyond. *Curr Protoc* **1**, e323 (2021). https://doi.org/10.1002/cpz1.323

3 Manni, M., Berkeley, M. R., Seppey, M., Simao, F. A. & Zdobnov, E. M. BUSCO Update: Novel and Streamlined Workflows along with Broader and Deeper Phylogenetic Coverage for Scoring of Eukaryotic, Prokaryotic, and Viral Genomes. *Mol Biol Evol* **38**, 4647-4654 (2021). https://doi.org/10.1093/molbev/msab199
